# Structure-Based Deep Learning Framework for Modeling Human–Gut Bacterial Protein Interactions

**DOI:** 10.3390/proteomes13010010

**Published:** 2025-02-17

**Authors:** Despoina P. Kiouri, Georgios C. Batsis, Christos T. Chasapis

**Affiliations:** 1 Institute of Chemical Biology, National Hellenic Research Foundation, 11635 Athens, Greece; despoina.kiouri.99@gmail.com (D.P.K.); georgebatsis95@gmail.com (G.C.B.); 2 Laboratory of Organic Chemistry, Department of Chemistry, National and Kapodistrian University of Athens, 15772 Athens, Greece

**Keywords:** gut microbiome, host–bacteria interactions, deep learning, neural networks

## Abstract

**Background:** The interaction network between the human host proteins and the proteins of the gut bacteria is essential for the establishment of human health, and its dysregulation directly contributes to disease development. Despite its great importance, experimental data on protein–protein interactions (PPIs) between these species are sparse due to experimental limitations. **Methods:** This study presents a deep learning-based framework for predicting PPIs between human and gut bacterial proteins using structural data. The framework leverages graph-based protein representations and variational autoencoders (VAEs) to extract structural embeddings from protein graphs, which are then fused through a Bi-directional Cross-Attention module to predict interactions. The model addresses common challenges in PPI datasets, such as class imbalance, using focal loss to emphasize harder-to-classify samples. **Results:** The results demonstrated that this framework exhibits robust performance, with high precision and recall across validation and test datasets, underscoring its generalizability. By incorporating proteoforms in the analysis, the model accounts for the structural complexity within proteomes, making predictions biologically relevant. **Conclusions:** These findings offer a scalable tool for investigating the interactions between the host and the gut microbiota, potentially yielding new treatment targets and diagnostics for disorders linked to the microbiome.

## 1. Introduction

Currently, the entire gut microbiome (GM) is being considered as an essential organ and major regulator of the human body, estimated to comprise more than 1014 microorganisms, according to the publicly available genomic and proteomic microbiome databases [1]. These bacteria, viruses, fungi, archaea, and protists coexist and interact in a complex system [1]. The roughly 3 million genes in the GM encode enzymes that produce thousands of metabolites, while the human genome only contains about 23,000 genes [2]. The majority of the bacterial species of the GM form symbiotic relationships with the host and are thus are crucial for the maintenance of host homeostasis, since they play an integral part in the establishment and regulation of intestinal innate and adaptive immunity [3]. Besides their effect on the digestive system, these bacteria additionally influence lateral organs such as the liver, brain, and pancreas. Therefore, it is no surprise that gut dysbiosis has been linked to a variety of illnesses, including neurodevelopmental [4], inflammatory [5], metabolic [6], cardiovascular [7], autoimmune [8], and psychiatric diseases [9], as well as cancer [10]. Consequently, any alterations of this complicated symbiotic relationship between the intestinal flora and the host can promote the development and progression of gut-related pathological conditions.

Recent studies have focused on the identification of protein interactions between human and bacterial species. High-throughput yeast two-hybrid assays have been used to pinpoint interactions between the proteins of various bacterial species, including *Bacillus anthracis*, *Francisella tularensis*, *Yersinia pestis*, *Mycobacterium tuberculosis*, and human host proteins [11,12,13]. Additionally, mass spectrometry (MS) and cross-linking assays have been developed to study host–bacteria interactions in a more native environment between human and *Salmonella enterica*, *Acinetobacter baumanii*, and *Lactobacillus acidophilus* proteins [14,15,16]. More recently, Li et al. employed a specialized bifunctional amino acid (i.e., photo-ANA) to study protein interactions between *Salmonella enterica serovar Typhimurium* and human proteins [17]. Considering the plethora of challenges linked to experimental approaches for unlocking the mysteries of the human gut microbiota, computational strategies have emerged as a first step towards addressing the complexity of this inter-species dynamic system.

In the early days, protein interaction prediction was performed through inferring domain–domain interactions (DDIs) from known protein–protein interactions (PPIs), assuming that if two proteins contain two interacting domains, they themselves are interacting too. At first, the DDI prediction was based on statistical approaches, such as Association Method and Maximum Likelihood Estimation [18]. Afterwards, optimization algorithms that pinpoint the minimum number of DDIs that satisfy a given PPI network (PPIN) like Linear Programming [19] and Genetic Algorithm [20] were developed. Later on, Machine Learning (ML)-based methods, such as Random Forest (RF) [21], were also used to predict PPIs based on domain information. The most recent approach of this type is based on Graph Theory [22].

Additionally, docking algorithms, such as HADDOCK [23], ClusPro [24], ZDOCK [25], LightDock [26], and InterEvDock [27], were used to predict PPIs by spatially orienting two proteins to find a potential binding site. Accumulated sequence and structure information of known PPIs is used in template-based computational techniques for PPI prediction and structural model construction [28,29]. Nowadays, the rapid evolution of artificial intelligence (AI) algorithms has led to significant advancements in computational techniques for PPI prediction. These AI methods are divided into two major categories, sequence-based [30,31,32] and structure-based [33,34,35].

Given the current state of knowledge, there is a scarcity of experimental studies that have successfully identified interactions between proteins from the bacteria of the GM and the human host, despite the presence of public databases containing experimental data on interactions between bacterial species and humans. This research gap may impede our understanding of how imbalances in the relationship between GM bacteria and humans contribute to the development of diseases. To obtain a better idea of the experimental data availability, an experimentally validated pan-human–bacterial protein interaction network was calculated from data that were retrieved from public databases (i.e., HPIDB [36,37], IntAct [38], PHISTO [39] and MorCVD [40]). To this day, this network contains less than 20 thousand interactions. Nevertheless, the entire gut microbiome is thought to comprise 300 to 500 different bacterial species, so it is safe to say that the interactions between them and the host proteins are really understudied due to a lack of data. Furthermore, each proteome of each organism (either bacteria or human) is not just a sum of the proteins encoded by its genetic code, but a rather complex collection of proteoforms. More specifically, every protein can be modified at any given time either before or after it is translated, resulting in a multitude of protein types where, even though, in some cases, they can share the same amino acid sequence, their function is not identical [41]. This study addresses these gaps by predicting a PPIN between gut bacterial and human proteins, using a novel structure-based deep neural network.

## 2. Materials and Methods

### 2.1. Deep Learning Architecture

**Figure 1:**
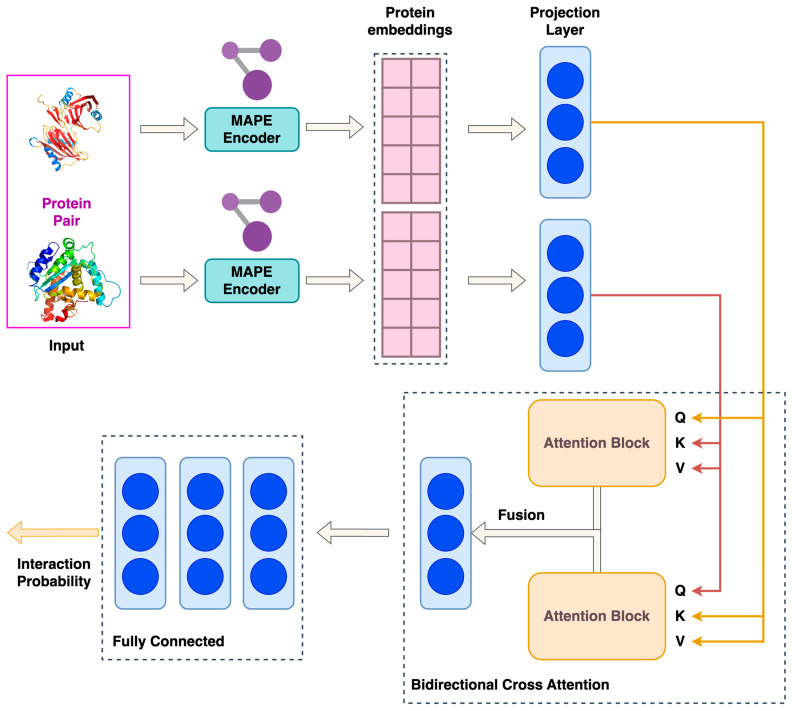
The overall DL model architecture.

The deep learning (DL)-based framework that was utilized for PPI prediction consists of three basic modules: (1) a graph-based structural protein embedding calculator; (2) a bi-directional attention-based embedding fusion layer; and (3) a PPI classifier. This DL architecture uses as input a pair of protein structures that is encoded in a pair of numerical representations through the graph-based embedding calculator. Next, the representation pair is aggregated via the attention-based fusion embedding layer, and a single protein pair embedding is generated. Finally, the last module of the framework performs the classification of each protein pair as either interacting or non-interacting through a series of fully connected layers. The overall model architecture is presented in Figure 1.

#### 2.1.1. Protein Embedding Calculator

The preprocessing stage of the embedding calculation involves the representation of each protein as a heterogeneous graph with nodes corresponding to individual amino acids. It should be noted that as the graph representation converts the protein structure into a graph to be processed, it can only account for the proteoforms that adopt different conformations due to changes in the amino acid sequence. Three distinct edge types are included in each protein graph: (1) sequence-based edges that connect consecutive amino acids in the primary sequence; (2) radial distance-based edges, which connect amino acids within a predefined spatial threshold of less than 10 Å in the 3D structure (Cα-Cα distance); and (3) k-nearest neighbor (k-NN) edges, connecting each amino acid to its k-nearest neighbors based on spatial proximity in the protein structure. Protein embeddings were then calculated using a pre-trained variational autoencoder (VAE) model, encapsulated in the Masked Autoencoder for Protein Embeddings (MAPE) framework [42]. The VAE architecture consists of an encoder, which maps each protein graph to a latent vector using a vector quantization (VQ) layer, and a decoder that reconstructs the encoder input. In this study, the decoder was not used because only the latent representation (i.e., the protein embedding) was needed. Since the encoder module of VAE was pre-trained on 14,952 proteins from the STRING database [43], it was frozen during the model’s training process to further enhance the model’s focus on the layers downstream of the encoder. Furthermore, according to Wu et al., VAE has an outstanding generalization capacity and thus it can perform accurate embedding calculations even if it has not been trained to the structures of the input proteins [42]. After its calculation, the latent representation is then fed through the VQ layer which transforms the continuous latent vector into a limited set of discrete prototypes, known as the microenvironment codebook. This codebook consists of a fixed number of embedding vectors, each corresponding to a unique structural microenvironment frequently encountered across various proteins.

#### 2.1.2. Bi-Directional Cross-Attention Module

The Bi-directional Cross-Attention module combines the embeddings of the two input proteins ($P_{1}$, $P_{2}$) into a unified pair representation. First, the two vectors are projected to two parallel trainable projection layers of dimensionality of 256 ($P_{1}^{\prime }$ and $P_{2}^{\prime }$) for computational efficiency and alignment with the attention mechanism, and these two vectors are passed through the Bi-directional Cross-Attention module. The core mechanism of this process is the attention mechanism described in the following equation (Equation (1)) [44]:

(1) $$ATN\left(q,k,v\right)=softmax\;(\frac{qk^{T}}{\sqrt{d_{h}}})v$$

Equation (1) is the of the attention matrix, where q,k,v are the query, key, and value matrices, respectively, $d_{h}$ is the dimensionality of each attention head, and *softmax* is the activation function that ensures that the attention weights sum to 1.

Multi-head attention is applied, and the input is split into h heads (Equation (2)):

(2) $$MH\left(q,k,v\right)=Concat({head}_{1},\;\ldots ,\;{head}_{h})W_{o}\;where\;{head}_{i}=ATN(q_{i},\;k_{i},\;v_{i})$$

Equation (2) shows the calculation of multi-head attention.

In this work, this mechanism is applied in both directions: $P_{1}^{\prime }\;$attends to $P_{2}^{\prime }$ and $P_{2}^{\prime }\;$attends to $P_{1}^{\prime }$. Additionally, the attended sequence for each protein embedding is combined with the initial input (Equation (3)):

(3) $$F_{1}=\;P_{1}^{\prime }\;+\;MH\left(P_{1}^{\prime },P_{2}^{\prime },P_{2}^{\prime }\right)\;and\;F_{2}=\;P_{2}^{\prime }\;+\;MH\left(P_{2}^{\prime },P_{1}^{\prime },P_{1}^{\prime }\right)$$

Equation (3) shows the bi-directional implementation of multi-head attention.

Finally, $F_{1}$ and $F_{2}$ are concatenated and protected to a linear layer of dimensionality of 256 to obtain the representation of the protein pair.

#### 2.1.3. Fully Connected Layers—Classification Process

The fused embedding was then passed through a series of fully connected layers to predict the likelihood of interaction of each protein pair. The intermediate layer dimensions were 256 and 128, respectively, with ReLU activation functions applied after each layer. Dropout regularization was applied with rates of 0.5 and 0.3 in the first and second fully connected layers, respectively. The final layer produced a scalar output, representing the interaction score between P1 and P2. Given the imbalance between interacting and non-interacting protein pairs in the dataset, the focal loss function was employed to mitigate class imbalance. The focal loss function modifies the standard binary cross-entropy loss by introducing a modulating factor (1 − p_t_)^γ^, which emphasizes hard-to-classify samples. The focal loss (Equation (4)) is defined below:

(4) $$L_{FL}\left(p_{t}\right)=a_{t}\;{\left(1-p_{t}\right)}^{\gamma }log(p_{t}),$$

Equation (4) shows the focal loss function, where $p_{t}$ is the predicted probability for the true class, $a_{t}$ is the weighting factor for class imbalance, and γ is the focusing parameter (γ = 2).

This function allows the model to focus on misclassified or difficult samples (i.e., the interacting pairs that are far fewer than the non-interacting pairs), thereby improving overall performance in imbalanced datasets.

#### 2.1.4. Training Process

The model parameters were optimized using the Adam optimizer with an initial learning rate of 0.001. To further refine the learning process, a learning rate scheduler was employed, which reduced the learning rate when no improvement in validation loss was observed. Early stopping was incorporated with a patience of five epochs to prevent overfitting, and the maximum epoch number was set to 500. During training, the interaction prediction model utilized mini-batches of size 256.

### 2.2. Evaluation Metrics

For the classification assessment of protein interactions within the dataset, a robust evaluation framework encompassing basic error metrics and composite metrics was established. The essential components of the performance evaluation are the number of correctly classified samples and Type I and Type II errors [45], i.e., true positives (TPs), true negatives (TNs), false positives (FPs), and false negatives (FNs). These metrics were derived from the comparison of the model’s predictions against the labels that corresponded to reality. Macro-averaged precision, recall, and F1-score were calculated to provide an overall picture of the classifier’s performance across all classes. These metrics treat all classes equally, effectively averaging the class-wise metrics. Macro-averaging is particularly useful in imbalanced classification scenarios as it mitigates the effect of a model being heavily biased towards the majority class. The specific calculation involves obtaining precision (PREC), recall (REC), and F1-score (F1) for each class, as indicated in Equations (5)–(7), and then averaging those scores, giving an equal weight to each class in the calculation.

(5) $$PREC=\;\frac{TP}{TP+FP}$$

Equation (5) shows the precision: the ratio of correctly predicted positive observations to the total predicted positives.

(6) $$REC=\;\frac{TP}{TP+FN}$$

Equation (6) shows the recall: the ratio of correctly predicted positive observations to all observations in actual class.

(7) $$F1=\;2\frac{Precision*Recall}{Precision+Recall}$$

Equation (7) shows the F1-score: the harmonic mean of PREC and REC.

Focusing on the classification of the interacting class (the minority class of interest), precision, recall, and F1-score were calculated. These metrics provide a detailed view of the model’s performance on this particular class, as an accurate performance for the interacting class is paramount. The Matthews Correlation Coefficient (MCC) (Equation (8)) was calculated as a measure of the correlation between the actual and predicted classifications, taking into account true positives, true negatives, false positives, and false negatives.

(8) $$MCC=\;\frac{TP*TN-FP*FN}{\sqrt{\left(TP+FP\right)*(TP+FN)}*\left(TN+FP\right)*(TN+FN)}$$

Equation (8) shows the Matthews Correlation Coefficient (values ranging between −1 and 1), where 1 represents a perfect prediction, 0 is not better than random prediction, and −1 is a complete disagreement between true and predicted classes.

Balanced ACC (ACC_B_) was calculated as the average of recall across all classes, which is equivalent to the arithmetic mean of the sensitivity and specificity. This measure mitigates the impact of class imbalance by accounting for performance on both the majority and minority classes. The average PREC (AP) score was calculated as the average precision across all thresholds in the precision–recall curve, effectively summarizing the model’s performance across various operating points. AP values range between 0 and 1, with a higher value indicating better performance.

The Receiver Operating Characteristic (ROC) curve, a graphical representation of a model’s prediction capability by plotting the true positive rate (TPR) (Equation (9)) versus the false positive rate (FPR) (Equation (10)) at various threshold values, was also calculated. Finally, the Area Under the Curve (AUC) score that quantifies the model’s overall ability to distinguish between positive and negative classes was computed. A higher AUC score suggests superior model performance and generalization capabilities. Finally, the REC value obtained at an operating point where the precision is at least 0.5 was measured as REC@PREC = 0.5.

(9) $$TPR=\;\frac{TP}{TP+FN}$$

Equation (9) shows the true positive rate.

(10) $$FPR=\;\frac{FP}{FP+TF}$$

Equation (10) shows the false positive rate.

## 3. Results

### 3.1. Dataset Construction—Model Training

First, all publicly available experimental pan-human–bacterial PPI data, which contained 19,686 interactions between 5714 bacterial and 4287 human proteins, were retrieved from four public databases, HPIDB [36,37], IntAct [38], PHISTO [39], and MorCVD [40] (original dataset). Another more inclusive PPI dataset that contained interactions from six widely used interaction databases (i.e., IntAct [38], MINT [46], DIP [47], HPRD [48], BioGRID [49], and SIFTS [50,51]) was also obtained. This inclusive extended PPI dataset contains 1,081,401 PPIs, out of which 330,530 are human inter-species PPIs and 750,871 are inter- and intra-species interactions of different organisms, including bacteria, viruses, plants, and animals. In this extensive dataset, 13 pairs of PPIs between the host and gut bacterial proteins were identified, but none of them consisted of proteoforms of the same gene. All the proteins of the original and larger dataset were also mapped to their protein structures using the AlphaFold database API [52,53] in order to eliminate the factor of differences in structural quality between different proteins. For the construction of the positive dataset, both the original and the larger PPI datasets were then filtered, and only the interactions where both participating proteins were matched to available protein structures were kept. Next, a negative dataset that contained proteins that do not interact was constructed using only human proteins that are solely present in different organs of the human body, and at the same time, their domains (i.e., Pfam domains [54]) do not interact. The complete human proteome was retrieved from UniProt Proteomes, and the tissue topology of every individual protein was then obtained from the Human Protein Atlas [14]. Finally, a dataset (gold-standard dataset) containing 17,278 experimentally supported DDIs from PDB complexes was retrieved from the 3did database [22,55]. Additionally, these PPIs were filtered and only those that did not exist in the positive dataset or the available human interactome were kept.

The positive and negative datasets were combined into one large-scale PPI and non-PPI dataset that was then divided into three datasets: the training dataset (60%), the validation dataset (20%), and the test dataset (20%). The division was performed in such a way that all three subsets had same class distribution (Table 1). From the training dataset, it is evident that there is an imbalance between interacting and non-interacting protein pairs.

For overfitting prevention, the model training ended at 13 epochs because validation loss was not reduced.

### 3.2. Model Evaluation Based on Validation Dataset—Decision Threshold Calculation

**Figure 2:**
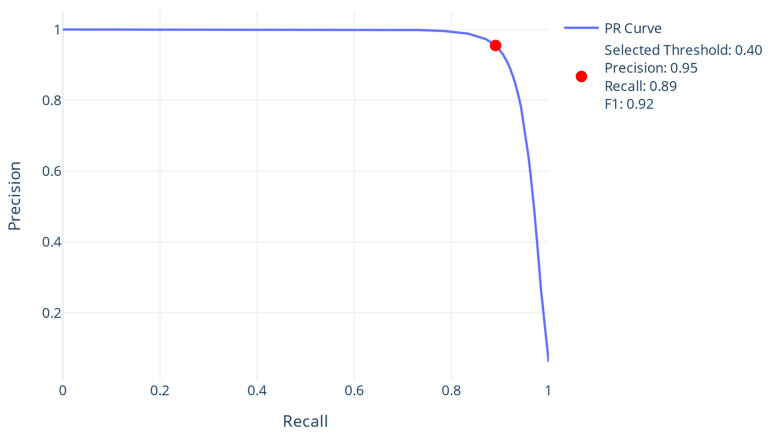
The PREC-REC curve using the validation dataset. The red point corresponds to the optimal threshold (0.4) that maximizes F1-score. The precision, recall and F1-score are the performance metrics of this threshold.

Next, the validation dataset was used for the selection of the optimal decision threshold (DT). In this case, the best DT was chosen so that the binary F1-score was maximized (Table 2). The optimal decision threshold is visualized on the PREC-REC curve of the validation dataset in Figure 2.

### 3.3. Model Testing

**Figure 3:**
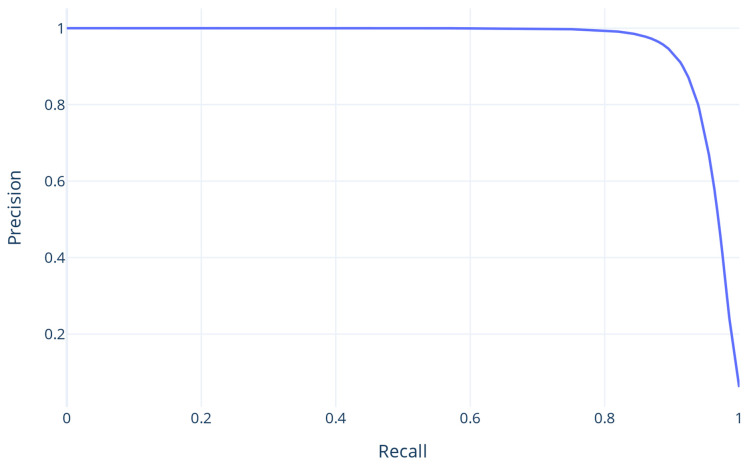
The PREC/REC curve.

**Figure 4:**
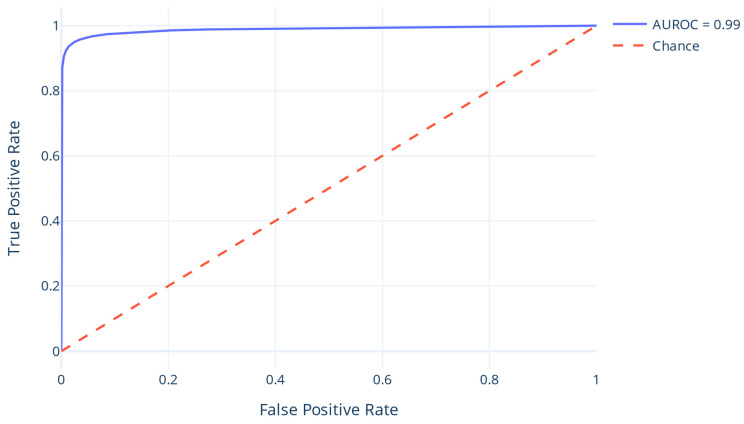
The ROC curve.

Using the chosen DT, the test dataset was used for the final evaluation. To evaluate the prediction, the confusion matrix that demonstrates the proportion of accurate and inaccurate predictions per class was calculated (Table 3). The model was also evaluated using ACC, F1, PREC, and REC (Table 4) as well as the PREC/REC (Figure 3) and ROC curve, whose Area Under Curve (AUC) is 96 (Figure 4). When applied to the test set, the model exhibited evaluation metrics equivalent to the validation set, demonstrating its generalization capability. This similarity between the test and the validation outcomes suggests that the model effectively captures the underlying patterns within the data, minimizing overfitting and validating its robustness in unseen scenarios.

### 3.4. Model Deployment

Since, in this case, the model deployment of choice is the prediction of the protein interaction network between human gut proteins and bacterial proteins, these proteins were also retrieved. The human proteome was filtered using only the labels for gut (i.e., ‘oral mucosa’, ‘salivary gland’, ‘esophagus’, ‘stomach 1’, ‘stomach 2’, ‘duodenum’, ‘small intestine’, ‘colon’, ‘rectum’, ‘liver’, ‘gallbladder’, ‘pancreas’, ‘appendix’, ‘smooth muscle’, ‘adipose tissue’, ‘soft tissue 1’, ‘soft tissue 2’) and brain topologies (i.e., ‘caudate’, ‘cerebellum’, ‘cerebral cortex’, ‘hippocampus’, ‘hypothalamus’, ‘pituitary gland’, ‘choroid plexus’, ‘dorsal raphe’, ‘substantia nigra’) from the Human Protein Atlas [56], and then only the entries with available structures were kept. The inclusion of brain-related data in the model’s application was driven by the growing recognition of the gut–brain axis as a critical area of research [57]. This bidirectional communication network between the gut microbiota and the central nervous system plays a significant role in regulating neurological and psychological health [58,59].

From the Human Gut Microbiome Atlas [56,60], bacterial strains that are labeled as ‘Healthy’ were chosen and were then mapped to their respected proteins. Subsequently, all the proteins that were included in each Proteome ID were retrieved using the Proteins API of the European Bioinformatics Institute (EBI) [61], and only the entries with available protein structures were kept. Additionally, to properly address proteome complexity, the proteoforms for both human and bacterial proteins were included in this study. The term ‘proteoform’, established by Smith et al. [41], is used to describe ‘all of the different molecular forms in which the protein product of a single gene can be found, including changes due to genetic variations, alternatively spliced RNA transcripts and post-translational modifications’. In this study, all the proteoforms that are documented as separate UniProt entries sharing a gene name are included.

**Figure 5:**
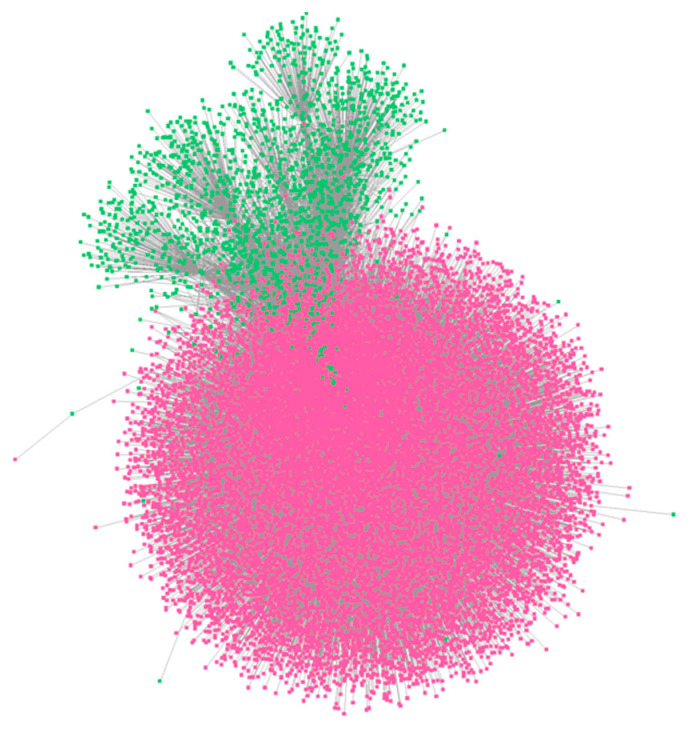
A part of the predicted protein network between human (pink) and gut bacterial (green) proteins with prediction interaction score equal to or greater than 0.99. The network visualization was performed using Cytoscape (version 3.9.1) [62].

The total numbers of human and gut bacterial proteins used in the prediction task were 24,345 and 100,945, respectively, resulting in a total of 2,457,506,025 protein pairs. The graph representations of the proteins were used as model input. Such graphs encode the structural relationships between amino acids that were represented as nodes, as mentioned in the preprocessing steps above. Batch processing was utilized to improve computational efficiency and enable large-scale predictions. During inference, the trained model was loaded, and predictions were generated using a sigmoid activation function, which extracts probabilities from the model’s output. These probabilities represent the confidence of interaction between protein pairs. During model development, a threshold of 0.4 was employed based on balanced predictive performance metrics derived from the unseeded test subset. This threshold was selected to capture a wide range of potential PPIs while maintaining an equilibrium between sensitivity and specificity, thereby ensuring the model’s generalizability across diverse scenarios. In contrast, a higher threshold of 0.99 was applied specifically for gut microbiome predictions. This choice was driven by two primary considerations: first, to prioritize high-confidence, high-probability interactions and enhance the reliability of predictions, and second, to address the practical constraint of space limitations associated with reporting large-scale PPI datasets. As a result, only those pairs whose predicted probabilities exceeded the threshold are assigned as interacting (i.e., only the protein pairs with a prediction probability of equal to or greater than 0.99 are considered to interact). This assumption led to a total of 16,106,277 predicted PPIs between 19,054 human and 1886 bacterial proteins, representing approximately 0.6% of the total protein pairs processed. The predicted interactions were used to construct a PPI network, as illustrated in Figure 5. Due to computational limitations, only a part of this network was illustrated. More information about the predicted interactions is presented in Supplementary Materials S1. Finally, an analysis to identify if the proteoforms of the same protein interact with the same proteins was performed. In Table 5, results that are indicative of the analysis mentioned above are represented. From this analysis, it is evident that there are different interacting partners between proteoforms of the same protein.

## 4. Discussion

The results of this study demonstrate the efficacy of a novel DL-based framework in predicting PPIs between human and gut bacterial proteins using structural data. The significance of this research lies in the successful application of advanced computational techniques to a complex biological system, where traditional experimental methods have faced limitations. This work addresses the existing gap in deciphering the unknowns of the gut microbiome–host interaction network, which is necessary for understanding the role of the microbiome in health and disease.

The implementation of a graph-based protein representation was a key factor in the model’s success, as it allowed for the accurate capture of the structural relationships between amino acids. By leveraging the VAE for protein embedding calculation, the model could efficiently process structural data, enabling the detection of interaction patterns that would otherwise remain obscured by conventional sequence-based approaches. This method demonstrates superior performance, particularly in identifying interactions in large-scale datasets with significant class imbalance, a common issue in PPI prediction tasks. The use of focal loss for handling the class imbalance further enhanced the model’s robustness, allowing it to prioritize harder-to-classify interacting pairs, which are often underrepresented in existing datasets. Unlike other methods that generate random pairs for non-interactions without biological filtering, this model incorporates biological knowledge to refine the negative dataset, further enhancing its accuracy.

One of the notable strengths of the model lies in its Bi-directional Cross-Attention fusion layer, which incorporates an attention mechanism that aggregates the embeddings of protein pairs. This method offers a more nuanced fusion of features compared to traditional concatenation techniques, by dynamically recognizing the importance of different features and portraying how one protein attends to the other. The model’s generalization ability, as evidenced by the high AUC score and consistent performance across both validation and test datasets, suggests that the model does not overfit and can effectively be applied to unseen data. Therefore, this framework can be applied to other biological contexts, including other distinct host microbiomes, such as the oral, nasal, and skin microbiomes that have all emerged as crucial regulators of the host’s health [63,64,65].

From a biological perspective, another important asset of this model is its ability for proteoform inclusion in the predicted networks. As mentioned above, proteoforms are the source of variance in proteomes that make them far more complex than genomes and transcriptomes [41]. Since the contribution of proteoforms in proteome complexity is shown in the form of different proteins for a single gene, this model’s prediction outcome is very close to reality as it uses all the available protein structures that are included in each proteome, and not just the reference protein for each gene. The percentage of proteoforms in humans is higher than the corresponding percentage of gut bacteria. This outcome is expected since proteome complexity is higher, and events of post-translational modification are more common in eucaryotes than procaryotes [60]. The proteoform analysis revealed that although proteoforms of the same protein share some protein interactors, each proteoform has some additional unique interactors. This finding is very important as it shows that members of the same proteoform not only have differences in terms of sequence and even structure, but they also interact with different proteins. That being the case, it is pivotal that they are incorporated in proteome studies because they directly contribute to proteome complexity.

The scarcity of experimentally validated human–gut bacterial PPIs has been a significant setback in understanding the contributions of this interplay in gut-related disease emergence. Therefore, this study opens new avenues for the discovery of previously unknown protein interactions, that could serve as novel therapeutic targets and biomarkers.

The predictive PPI network generated by this study represents a valuable resource for further experimental validation. The next steps for experimental validation of the key interactions identified, particularly those involving proteoforms with limited characterization, will focus on integrating advanced proteomics techniques with functional assays. High-resolution MS-based approaches [66], such as cross-linking MS [67] or co-immunoprecipitation coupled with MS [68], can be employed to validate interactions in biologically relevant contexts and provide structural insights into proteoform-specific interactions. Additionally, cellular localization studies using fluorescent tagging [69] and live-cell imaging [70] can confirm the physical proximity of interacting proteins under physiological conditions.

Despite the significant strengths of this model, there are certain limitations that should be acknowledged. The model’s reliance on structural data restricts its applicability to proteins with known or predicted structures, excluding a portion of the proteome from analysis, including proteins with intrinsic disorder. In contrast, sequence-based methods can be applied to any protein for which sequence data are available, irrespective of structural information. Additionally, the DL architecture used in this framework is computationally demanding. The graph-based embeddings and the attention mechanism require computational resources, which may limit the usage of the model in real-time applications or in environments with limited resources. Simpler methods, such as Random Forest or lighter DL architectures, may offer advantages in scenarios where computational efficiency is prioritized. Although the model excels at handling class imbalance through the focal loss function, it may still encounter challenges in extreme cases where positive samples (i.e., protein interactions) are exceedingly scarce. Finally, the performance of the model is closely tied to the quality of the input data, particularly the structural data provided by the AlphaFold database and the embeddings from the VAE. Any errors or inaccuracies in these data sources may propagate through the model, potentially affecting its predictions.

## 5. Conclusions

This study presents a novel DL framework designed to predict PPIs between human and gut bacterial proteins based on structural information. By leveraging graph representations of protein structures and integrating them through attention-based fusion and VAE-generated embeddings, the model achieves a high level of prediction accuracy. The proposed method successfully addresses the challenges posed by data imbalance in PPI datasets and demonstrates robustness across diverse protein pairs.

Given the scarcity of experimental data concerning interactions between human and gut bacterial proteins, this framework not only fills a critical gap in existing knowledge but also establishes a scalable method for identifying novel interactions. The predictive network derived from this study presents a valuable resource for further biological investigations and experimental validation, potentially contributing to the understanding of gut microbiome–host interactions and their implications in human health and disease. Additionally, since this prediction method includes proteoforms, it can be utilized as a tool for identification of protein indicators of disease, remission, response to therapy, and drug target, in cases where the protein of interest is a proteoform of a common protein. Overall, this study showcases the potential of DL in advancing computational biology and bridging the gap between theoretical prediction and experimental validation in PPI studies.

## Figures and Tables

**Table 1:** Number of samples per dataset subset and category (i.e., positive, negative).

Datasets	PPIs	Positive	Negative
Train	10,681,662	654,604	10,027,058
Validation	2,670,416	163,651	2,506,765
Test	3,338,020	204,564	3,133,456
Total Number of Samples:	16,690,098		

**Table 2:** Performance metrics on validation dataset.

Performance Metrics	Values
Threshold	0.40
PREC (Macro)	0.97
REC (Macro)	0.94
F1 (Macro)	0.95
PREC (Interaction)	0.95
REC (Interaction)	0.89
F1 (Interaction)	0.92
MCC	0.91
ACC_B_	0.94
AP	0.96
AU-ROC	0.98
REC@PREC = 0.5	0.96

**Table 3:** Confusion matrix for test dataset.

	Predicted Negative	Predicted Positive
**Actually Negative**	3,124,742	8714
**Actually Positive**	22,647	181,917

**Table 4:** Performance metrics on test dataset.

Performance Metrics	Values
PREC (Macro)	0.97
REC (Macro)	0.94
F1 (Macro)	0.95
PREC (Interaction)	0.95
REC (Interaction)	0.88
F1 (Interaction)	0.92
MCC	0.91
ACC_B_	0.94
AP	0.95
AU-ROC	0.98
REC@PREC = 0.5	0.96

**Table 5:** The predicted protein interactions of 5 human and 5 bacterial proteoform families.

Human Proteins			
Gene	Protein Name	Number of Proteoforms	Number of Common Interactions
FOXP4	Forkhead box protein P4	2	1/2 (50%)
TNPO2	Transportin-2	4	53/106 (50%)
ME2	NAD-dependent malic enzyme, mitochondrial	9	259/2040 (12.7%)
HARS1	Histidine--tRNA ligase	11	268/35,590 (0.7%)
**Bacterial Proteins**			
**Gene**	**Protein Name**	**Number of Proteoforms**	**Number of Common Interactions**
rpsM	Small ribosomal subunit protein uS13	19	13/40 (32.5%)
xseB	Exodeoxyribonuclease 7 small subunit	2	32/32 (100%)
aroK	Shikimate kinase 1	3	1/3 (33.3%)
thiD	Hydroxymethylpyrimidine/phosphomethylpyrimidine kinase	2	2400/67,832 (3.5%)

## Data Availability

The code for this paper is available in the following GitHub repository: https://github.com/c3biolab/struct_ppi_pred, accessed on 7 October 2024. The data presented in this study are available from the corresponding author on request.

## Supplementary Materials

The following supporting information can be downloaded at https://www.mdpi.com/article/10.3390/proteomes13010010/s1: Supplementary Materials S1: The binary file containing protein interactions between human and gut bacterial proteins in UniProt IDs, with a prediction probability threshold of 0.99. The following supporting information can be downloaded at https://doi.org/10.5281/zenodo.14780446: Supplementary materials S2: Healty_Bac_predictions.zip: A .json file for every human protein with its bacterial interactors and interaction probability scores, as well as a binary file with all the PPIs in tab-separated format.
